# Does Organic Farming Increase Raspberry Quality, Aroma and Beneficial Bacterial Biodiversity?

**DOI:** 10.3390/microorganisms9081617

**Published:** 2021-07-29

**Authors:** Daniela Sangiorgio, Antonio Cellini, Francesco Spinelli, Brian Farneti, Iuliia Khomenko, Enrico Muzzi, Stefano Savioli, Chiara Pastore, María Teresa Rodriguez-Estrada, Irene Donati

**Affiliations:** 1 Department of Agricultural and Food Sciences, University of Bologna, 40127 Bologna, Italy; daniela.sangiorgio2@unibo.it (D.S.); antonio.cellini2@unibo.it (A.C.); enrico.muzzi@unibo.it (E.M.); stefano_savioli@fastwebnet.it (S.S.); chiara.pastore@unibo.it (C.P.); maria.rodriguez@unibo.it (M.T.R.-E.); irene.donati@zespri.com (I.D.); 2 Research and Innovation Center, Fondazione Edmund Mach, 38010 San Michele all’Adige, Italy; brian.farneti@fmach.it (B.F.); iuliia.khomenko@fmach.it (I.K.); 3 Zespri Fresh Produce, 40132 Bologna, Italy

**Keywords:** *Rubus idaeus* L., VOCs, microbiome, volatilome, *Gluconobacter*, anthocyanins

## Abstract

Plant-associated microbes can shape plant phenotype, performance, and productivity. Cultivation methods can influence the plant microbiome structure and differences observed in the nutritional quality of differently grown fruits might be due to variations in the microbiome taxonomic and functional composition. Here, the influence of organic and integrated pest management (IPM) cultivation on quality, aroma and microbiome of raspberry (*Rubus idaeus* L.) fruits was evaluated. Differences in the fruit microbiome of organic and IPM raspberry were examined by next-generation sequencing and bacterial isolates characterization to highlight the potential contribution of the resident-microflora to fruit characteristics and aroma. The cultivation method strongly influenced fruit nutraceutical traits, aroma and epiphytic bacterial biocoenosis. Organic cultivation resulted in smaller fruits with a higher anthocyanidins content and lower titratable acidity content in comparison to IPM berries. Management practices also influenced the amounts of acids, ketones, aldehydes and monoterpenes, emitted by fruits. Our results suggest that the effects on fruit quality could be related to differences in the population of *Gluconobacter*, *Sphingomonas*, *Rosenbergiella*, *Brevibacillus* and *Methylobacterium* on fruit. Finally, changes in fruit aroma can be partly explained by volatile organic compounds (VOCs) emitted by key bacterial genera characterizing organic and IPM raspberry fruits.

## 1. Introduction

Raspberry (*Rubus idaeus* L.) is a highly valuable crop with a large agricultural potential, both in Europe and worldwide. Raspberry consumer demand, as well as its cultivation, have been steadily increasing in the past 20 years, with a further acceleration since 2010 [1]. Russia is the top producing country, with a production volume of 165,800 tonnes accounting approx. for 20% of the world production [1]. Raspberry is also widely cultivated in other European (Serbia, Poland) and north-American (Mexico, USA) countries [1,2].

Hedonic characteristics and nutraceutical properties of raspberry are the main drivers of consumer appreciation. In fact, raspberry is rich in health-beneficial compounds, such as flavonols, catechins, ascorbic acid and ellagic acid derivatives [3,4]. Additionally, more than 300 volatile organic compounds (VOCs) have been reported as constituents of raspberry aroma [5], including alcohols, aldehydes, ketones, esters and terpenoids [6,7]. Among the main aroma-active compounds (i.e., those with low perception threshold compared to emission rates), C6 aldehydes (hexanal, hexenal isomers) and norisoprenoids (e.g., ionones) are responsible for grassy and floral notes of raspberry fruit aroma [8].

The organic farming system consists of a low-input crop management (EC regulation No. 834/2007) excluding synthetic pesticides and fertilizers for plant protection and nutrition [9]. In the last decade, organic food gained in popularity and the global market for certified organic products underwent a remarkable increase in sales (+72% 2009–2014) [10]. Berry fruits have followed this trend, with a rise in the organic production from around 30 tonnes in 2012 to 70 tonnes in 2018 [11]. However, the fulfilment of the increasing demand of organic raspberries faces several obstacles mining the growers profitability [12]. High costs are linked to the conversion of farms from integrated pest management (IPM) to the organic system and to obtaining of certifications, which are necessary for organic labelling, highly valued by consumers [2]. Additionally, organic raspberry farming has been found to be characterized by lower yields [12,13]. Furthermore, raspberry cultivation is affected by several pests and pathogens, including the insects *Resseliella theobaldi* (Diptera, Cecidomyiidae), *Lasioptera rubi* (Diptera, Cecidomyiidae), *Anthonomus rubi* (Coleoptera, Curculionidae), *Aphis idaei* (Hemiptera, Aphididae) and *Drosophila suzukii* (Diptera, Drosophilidae), the mite *Tetranychus urticae* (Acarina, Tetranychidae), the oomycete *Phytophtora fragariae* var. *rubi,* and the fungi *Leptosphaeria coniothyrium, Didymella applanata, Botrytis cinerea* and *Sphaerotheca macularis* [14]. Such pests and pathogens can compromise plant survival and/or fruit quality in the absence of adequate control measures [15,16]. In organic systems, control inputs, besides being characterized by high prices, are few and specialized literature regarding appropriate control methods is lacking [12]. On the other hand, growers profitability for organic food products is generally higher than IPM ones [12] due to the application of premium prices consumers are willing to pay [17]. Indeed, in developed countries, organic food is considered safer and healthier [18,19], as well as better for the climate and the environment [20] when compared to IPM grown one. Several studies observed higher contents of polyphenols, vitamins, carotenoids and ellagic acid, as well as fruit dry matter, sucrose, malic acid and minerals, in organic berries [21]. However, other studies came to contrasting conclusions in establishing causalities between improved nutritional properties and organic cultivation system which might be due to the numerous variables interacting with the final fruit quality.

Plants are known to host a great diversity of microbes, whose composition and functionalities can shape plant phenotype, performances and productivity [22,23]. The increasing access to genomic data has allowed a more in-depth investigation of the microbial communities (microbiomes) associated to the plant [24] and of their interaction with the host, pointing to a role of crop-associated microbes in agricultural performance and crop quality [25,26]. Although in the last few years several studies focused either on the microbial diversity present on various fruit species [27] or on the impact of root or foliar microbial applications on nutrient, flavonoid, organic acid contents and volatile compounds levels of fruits [28], a deep understanding of the influence of native fruit epiphytic microbiomes on fruit quality and on fruit volatilome building is still lacking.

Cultivation methods can influence the composition and structure of the plant microbiome [29] and differences observed in the nutritional quality of differently grown fruit [30] might be due to variations in the microbiome structure. Although dynamics of the soil microbiota under different cultivation methods have been extensively investigated [31,32,33], only a few studies focus on the influence of cultivation practices on fruit microbiomes [29]. Thus, the aim of this work was to comprehensively dissect the effect of organic or IPM cultivation on fruit microbiome, fruit quality characteristics and aroma. Contextually, differences of organic and IPM raspberry fruit-associated microbiomes were examined by next-generation sequencing (NGS) and bacterial isolates characterization in order to highlight the potential contribution of the resident microflora to fruit quality and aroma.

## 2. Materials and Methods

### 2.1. Plant Material and Cultivation Method

Raspberry fruit of the primocane ‘Enrosadira’ were harvested at the end of September 2019 in two different farms both located in the Cesena area (Emilia-Romagna region, Italy). The organic orchard was cultivated according to European Union (EU) regulation EC 889/2008. An IPM orchard was cultivated according to Agrointesa guidelines which comply with the national regulations (Legislative Decree 50/2012). In the latter orchard, IPM Epik^®^, Karate Zeon^®^, Laser^®^ and Signum^®^ were applied for the control of pests and fungal pathogens, using dose and application methods as suggested by the producers. The organic farm did not apply any kind of fertilization, whereas in IPM orchard, plants were fertigated weekly from April to September according to standard practices: calcium nitrate (125 kg/ha), magnesium nitrate (600 kg/ha), ammonium nitrate (100 kg/ha), potassium nitrate (250 kg/ha), potassium phosphate (200 kg/ha), monopotassium phosphate (300 kg/ha), nitric acid (52%, 1000 L/ha), iron (25 kg/ha) and microelements (15 kg/ha) in variable percentages depending on the phenological state.

To assess fruit quality, aroma and microbiome, fruits were randomly harvested at full ripe stage (i.e., when fruits were easily detached from the receptacle), put in cold boxes and immediately brought to the laboratory. Fruit volatiles and NGS analysis were performed on the same batch of fruits, consisting in three replicates each constituted by a pool of six fruits. The material was kept together for 24 h and subsequently halved for analysis processing.

### 2.2. DNA Extraction and Next-Generation Sequencing for Microbiome Analysis

To perform metagenomic analysis, three biological repetitions, consisting in a pool of three raspberry fruits, were washed in 20 mL MgSO_4_ 10 mM for 5 min under gentle agitation (70 rpm). Washing suspensions were frozen and stored at −80 °C. DNA was extracted according to the CTAB protocol [34], using the pellet obtained from centrifuging the washing solutions at 13,000× *g* for 10 min. DNA quality and quantity were measured by spectrophotometric quantification with a NanoDrop ND-8000 V1.1.1 spectrophotometer (Thermo Fisher Scientific, Dreieich, Germany). Bacterial V3-V4 regions were amplified with 16S Amplicon polymerase chain reaction (PCR) Forward = 5_TCGTCGGCAGCGTCAGATGTGTATAAGAGACAGCCTACGGGNGGCWGCAG and Reverse = 5_GTCTCGTGGGCTCGGAGATGTGTATAAGAGACAGGACTACHVGGGTATCTAATCC primers according to Illumina protocols and subjected to automated sequencing by BioFab research (Rome, Italy).

### 2.3. Bioinformatic Analysis

BBMap version 38.79 was used to remove low-quality reads using a quality-trim left and right ends before mapping with an average phred score ≥ 25 [35]. Sequences shorter than 170 bases were discarded. The sequences were analysed using the Qiime2 v. 2020.2.0 [36]. Qiime2 dada2 plugin was used to length trimming, denoising, chimera and PhiX removal. SILVA 16S rRNA sequences database, release 312 [37], with a 97% identity criterion was used to assign taxonomy to features. Taxonomic composition of organic and IPM raspberry fruit microbiomes was visualized by means of Krona chart created using Krona web application (https://github.com/marbl/Krona/wiki, accessed on 20 January 2021).

### 2.4. Cultural Dependent Characterization of Fruit Microbiome

Raspberry fruit washing obtained as above were serially diluted and plated on Luria-Bertani agar medium (Sigma Aldrich, St. Louis, MO, USA) amended with cycloheximide (100 µg/mL) to prevent fungal growth. Plates were incubated at 27 °C for 24 h. Colonies were randomly collected from the plates at the highest dilution. Isolates were stored at −80 °C in LB broth supplemented with 20% glycerol (*v*/*v*). DNA was isolated from bacterial isolates using GenElute Bacterial Genomic DNA kit (Sigma Aldrich, St. Louis, MO, USA), following manufacturer’s instructions. Genomic DNA patterns obtained by REP-PCR (Repetitive Element Palindromic PCR) and performed using primer BOXA1R [38], as described in [39], were visualized on a polyacrylamide gel [40].

Bacterial isolates were identified by 16s rRNA sequencing as follows: 16s rRNA gene extraction was amplified using Lac16S-for (5′-AATGAGAGTTTGATCCTGGCT-3′) and Lac16Srev (5′-GAGGTGATCCAGCCGCAGGTT-3′) primers, as described in [41]. The amplification product was delivered to Biofab Research Srl for sequencing. Sequences were compared with those available in http://www.ncbi.nml.nih.gov/BLAST, accessed on 15 January 2021.

Bacterial isolates were screened for several functional traits, according to the following protocols: indole-3-acetic-acid production—[42]; acetoin production—[43]; ammonia production—[44]; siderophores production—[45]; 1-aminocyclopropane-1-carboxylate (ACC) deaminase activity assay—[46].

### 2.5. Assessment of Fruit Quality Parameters

To measure soluble solids content (SSC) and fruit titratable acidity (TA), fruit juice was obtained homogenising berries and sieving them through a cloth. SSC of fruits was determined by digital refractometer (Atago-PAL1, Tokyo, Japan) and expressed as °Brix. Titratable acidity was determined manually by adding 0.1 M NaOH dropwise until reaching the titration end-point. Analyses were performed on nine replicates consisting of the juice from a pool of three berries each. Prior to homogenisation, fruit colour was determined using a CR-400 Chroma meter Colorimeter (Konica Minolta, Tokyo, Japan) on each berry. Three measurements were performed on the same fruit.

To determine the anthocyanins content, 3 g of homogenized fruit were suspended in 25 mL of methanol and incubated 24 h at room temperature. Samples were then centrifuged for 5 min at 4000× *g* and the supernatant was stored at −20 °C. Anthocyanin content was determined on the supernatant by using high-performance liquid chromatography (HPLC, Waters 1525, Waters, Milford, MA) at 520 nm according to [47].

Quantification was performed with the external standard method with the standards calibration curves (different concentrations were used to build the curve: 1–100 μg mL^−1^). Malvidin-3-glucoside chloride (Sigma Aldrich, St. Louis, MO, USA) was used as standard. The reliability of the quantification method was assessed by the R^2^ value of the standard and assessing that the concentration of each quantified compound was within the concentration range of the standard. Anthocyanin identification was performed according to [48].

### 2.6. Fruit Volatile Analysis

Raspberry fruit volatiles were analysed by gas chromatography–mass spectrometry (GC–MS). Each replicate (n = 3) consisted of a pool of three berries. Berries were put in a glass vial, closed with a lid equipped with a pierceable silicon cap and stored at −80 °C. Samples were taken out from −80 °C and put at −20 °C for 20 min before equilibration. After adding 1-octanol 0.05% (*v*/*v* in H_2_O) as internal standard, equilibration was performed for 20 min at 40 °C. Samples were then exposed to a 50/30-µm divinylbenzene/carboxen/polydimethyl siloxane (DVB Carboxen PDMS) Stable Flex 2 cm solid phase microextraction (SPME) fibre (Supelco, Bellefonte, PA, USA) for 40-min exposure. Afterwards, the SPME fibre was desorbed in a Shimadzu GC-MS-QP2010 Plus (Shimadzu, Tokyo, Japan) at 250 °C for 10 min in the split mode. The chromatographic separation of volatile compounds was performed on an RTX-WAX fused-silica capillary column (30 m × 0.25 mm i.d. × 0.25 µm) coated with polyethylene glycol (PEG) (Restek, Bellefonte, PA, USA). A three-step heating oven program was set: (1) 45 °C, 10 min; (2) 4 °C min^−1^ temperature increase to 200 °C; (3) 200 °C, 8 min. Helium was used as the carrier gas at a constant flow rate of 1 mL/min. A mass range from 33 to 400 *m*/*z* was scanned at a rate of 769 amu s^−1^. Mass spectra and linear retention indices (calculated according to the retention times of linear C8–C20 standard alkanes) were used for the identification of volatile compounds, based on the NIST/EPA/NIH Mass Spectral Database (NIST 08, National Institute of Standards and Technology, Gaithersburg, MD, USA) and the ChemSpider information resource (http://www.chemspider.com, accessed on October 2020). 

### 2.7. Bacterial Volatile Analysis by Proton-Transfer Reaction−Mass Spectrometry (PTR–MS) and in Silico Fruit Volatilome Assembly

10 mL commercial raspberry juice (Bocon, Italy) diluted with distilled water to 75%, adjusted to pH 7, were inoculated with 100 µL of freshly grown bacterial culture and incubated for 24 h. The experimental set foresaw three replicates for each bacterial isolate. Headspace volatiles were measured as described in [49] by using a proton-transfer reaction time of flight–mass spectrometer (PTR-ToF–MS) 8000 (Ionicon Analytik GmbH, Innsbruck, Austria). The drift tube conditions were as follows: 110 °C drift tube temperature, 2.8 mbar drift pressure, 428 V drift voltage, ion funnel (18 V). This leads to an E/N ratio of about 130 Townsend (Td), with E corresponding to the electric field strength and N to the gas number density (1 Td = 10−17 Vcm^−2^). The sampling time per channel of ToF acquisition was 0.1 ns, amounting to 350,000 channels for a mass spectrum ranging up to *m*/*z* = 400. The sample headspace was withdrawn through PTR-MS inlet with 40 sccm flow for 60 cycles resulting in an analysis time of 60 s/sample. Pure nitrogen was flushed continuously through the vial to prevent pressure drop. Each measurement was conducted automatically after 25 min of sample incubation at 40 °C and 5 min between each measurement was applied in order to prevent memory effect. All steps of measurements were automated by an adapted GC autosampler (MPS Multipurpose Sampler, GERSTEL) coupled to PTR-ToF–MS. The analysis of PTR-ToF–MS spectra proceeded as described in [49]. The array of masses detected with PTR-ToF–MS was reduced by applying noise and correlation coefficient thresholds. The first removed peaks that were not significantly different from blank samples; the latter excluded peaks with over 99% correlation, which mostly correspond to isotopes of monoisotopic masses. PTR-ToF–MS outputs of the isolates were employed to build the in silico fruit volatilome. Each virtual fruit sample was obtained by the sum of m/z emissions of the corresponding isolates, weighted for their NGS genus relative abundance. Multiple isolates belonging to the same genus were averaged before weighting m/z profiles assembled in this way were visualized by principal component analysis (PCA).

### 2.8. Statistical Analysis

Past software (Version 4.0) [50] was used for basic statistical functions and correlation canonical analysis. Student’s *t*-test was computed to investigate whether single VOCs, VOCs classes and quality parameters of organic and IPM managed samples were significantly different. The significance level of all analysis was *p* < 0.05. R (Version 1.1.463), together with the external package “mixOmics” [51], was used for PCA analysis and visualization employed in this work.

## 3. Results

### 3.1. Characterization of Raspberry Culture Dependent and Independent Bacteriome

**Figure 1:**
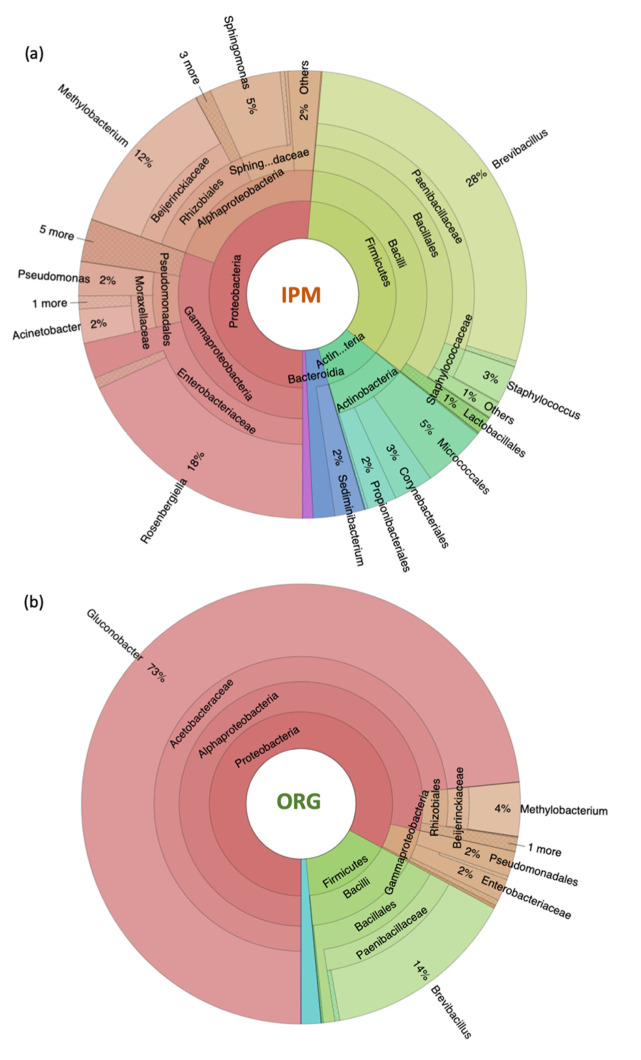
Krona chart showing bacterial community composition at phylum, order, class, family and genus level for (**a**) integrated pest management (IPM) and (**b**) organic raspberry fruits ‘Enrosadira’.

The composition of bacterial communities of raspberry fruit cultivated with organic or IPM strategies were assessed by NGS analysis of 16S rRNA gene. Sufficient sequencing effort was assessed (Figure S1). Community composition at the phylum level differed according to the cultivation method. Although both organic and IPM fruits were dominated by Bacilli and Alpha-proteobacteria, these phyla represented respectively 33% and 20% of total operational taxonomic units (OTUs) in IPM fruit, and 76 and 15% of total OTUs in organic fruits (Figure 1). Moreover, IPM fruits showed a higher bacterial biodiversity than organic ones (Shannon Index, H’ = 3.6 and 2, respectively) and also higher taxa evenness (J’ = 0.64 and 0.4, respectively; Table S1). The bacteriome of organic fruit was dominated by *Gluconobacter* spp. (73%), whereas IPM raspberries were colonized by several different taxa (*Brevibacillus* 14%, *Methylobacterium* 4%) (Figure 1). *Gluconobacter* spp. were absent in IPM fruits.

**Figure 2:**
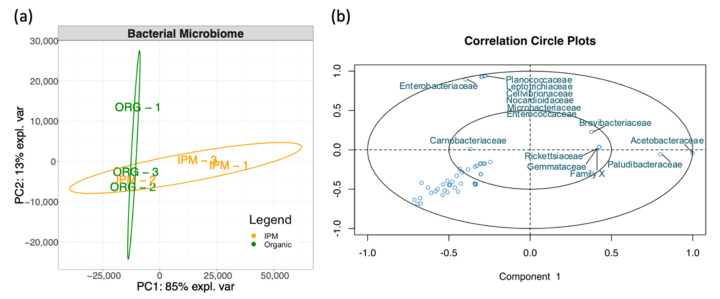
(**a**) Principal component analysis of bacterial families of raspberry fruits ‘Enrosadira’ cultivated with organic or IPM practices. Ellipses enclose confidence level = 95%. (**b**) Correlation circle plot with discriminating bacterial families is shown.

To visualize differences among the bacterial microbiomes of the two cultivation methods, PCA was performed according to bacterial families (Figure 2a). Organic produced fruit had more even composition of the bacterial community with berries discriminated only by PC2 (13% of the total variance). Despite the high uniformity of organic fruit, the analysis also highlighted that the differences in bacterial families between berries produced with organic and IPM practices provided only a partial discrimination of berries. Acetobacteraceae and Enterobacteriaceae were the first two families presenting the highest loading values for PC1 (85% of the expected variance) and thus mainly responsible for fruit discrimination (Figure 2b).

The total culturable bacterial population, expressed in log (colony-forming units (CFU)/fruit g), was higher in organic (6.1 ± 0.7) than IPM (5.6 ± 0.1). The analysis of culturable bacterial population indicated that IPM and organic fruit differentiated by few genera. *Rosenbergiella* was present only in IPM fruit, whereas *Tatumella*, *Klebsiella* and *Cronobacter* genera were found only in organic one (Table 1). IPM and organic unique bacterial isolates were screened for their in vitro plant growth promoting activities (Table 2). Indole-3-acetic-acid (IAA) producing bacteria, namely *Pantoea agglomerans* str. 2 and *Tatumella terrea*, were isolated only in organic fruit, whereas the sole acetoin-producing bacterium, *Enterobacter* spp. str. 2, was found on IPM fruit. All the isolates from organic fruit, but only one from IPM fruit, could release ammonia. Concerning siderophore production, *Klebsiella oxytoca* and *Enterobacter* spp. (str. 1 and 2) were the only species excreting this activity in organic and IPM fruit, respectively. Finally, none of the isolated strains neither in organic, nor IPM berries presented an ACC deaminase activity.

### 3.2. Fruit Quality Characteristics

**Figure 3:**
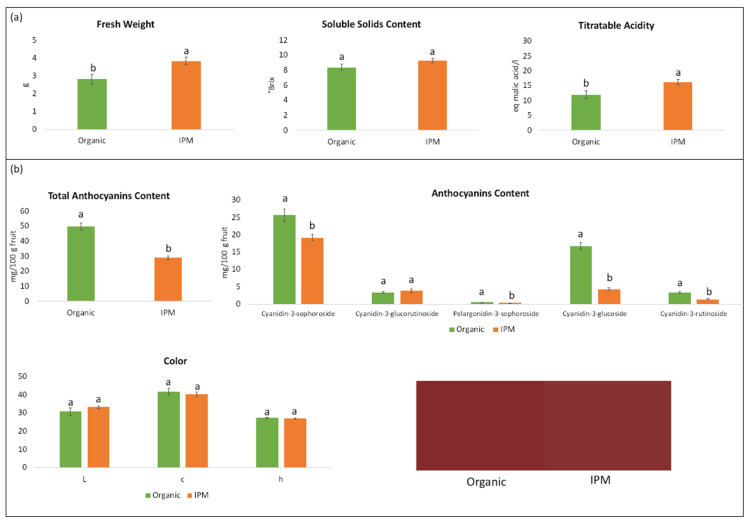
Main quality parameters (**a**), anthocyanidins content and color values (**b**) of raspberry fruit ‘Enrosadira’. Bars represent the mean ± SE. Different letters indicate significant differences between cultivation methods according to Student’s *t*-test at *p*-value < 0.05.

**Figure 4:**
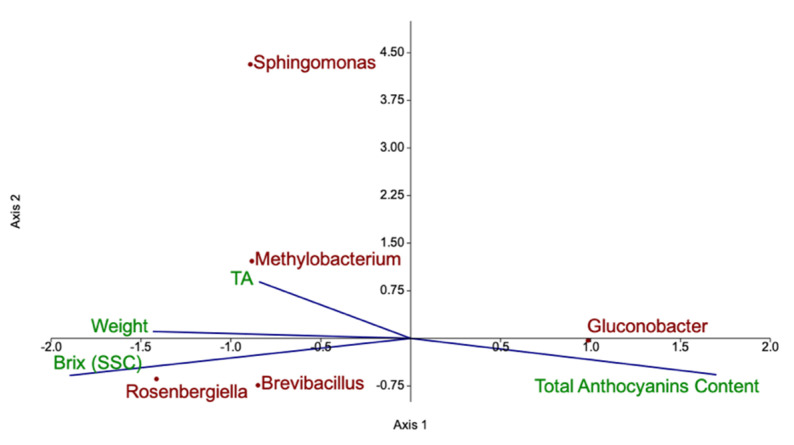
Canonical correlation analysis of bacterial genera discriminating organic from IPM grown fruit and quality parameters. Bacterial genera are coloured in brown, quality parameters in green.

Concerning sensorial quality traits, soluble solid content (expressed as °Brix) did not differ with respect to the cultivation method, whereas weight and titratable acidity (TA) were significantly lower in organically grown fruit (Figure 3a). Anthocyanins are the main compounds responsible for berry colour. Cyanidin-3-sophoroside was the most abundant anthocyanin in raspberry fruit, followed by cyanidin-3-glucoside (Figure 3b). Organic raspberries showed significantly higher concentration of all anthocyanins with the exception of cyanidin-3-glucorutinoside. The higher concentration of anthocyanins partially influenced berry colour, despite no significant differences were observed in any of the CIE coordinates (L × C × h). Canonical correlation analysis (CCA) was performed to investigate possible correlations between the bacterial genera harboured on fruit and the quality parameters (Figure 4). *Gluconobacter* positively correlated with total anthocyanin content, *Methylobacterium* and *Sphingomonas* with titratable acidity, whereas *Brevibacillus* and *Rosenbergiella* genera with soluble solid content.

### 3.3. Characterization of the Volatilome of Organic and Integrated Pest Management (IPM) Raspberry Fruits

**Figure 5:**
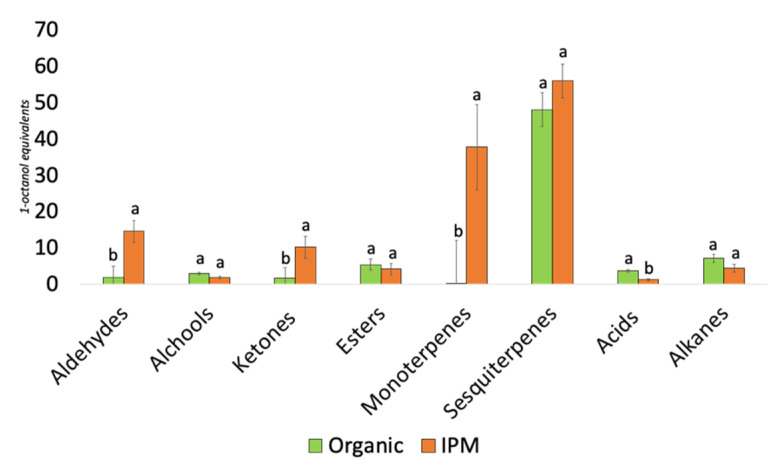
Abundance of volatile organic compound classes are showed for organic and IPM grown raspberry fruits ‘Enrosadira’. Bars represent the mean ± SE. Different letter indicate significant differences between cultivation methods according to Student’s *t*-test at *p*-value < 0.05.

The GC–MS raspberry volatile profile revealed 32 and 37 VOCs released by organic and IPM fruits, respectively. Single VOCs were grouped according to their chemical characteristics in the following classes: alkanes, acids, mono- and sesquiterpenes, esters, ketones, alcohols and aldehydes (Figure 5; Table 3).

**Figure 6:**
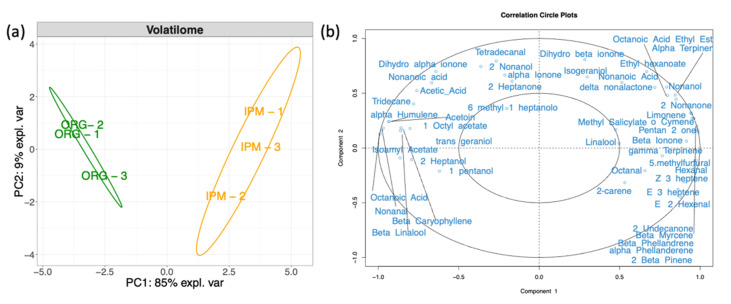
(**a**) Principal Component analysis of volatile organic compounds (VOCs) of raspberry fruits ‘Enrosadira’ cultivated with organic or IPM practices. Ellipses enclose confidence level = 95%. (**b**) Correlation circle plot with discriminating VOCs is shown.

The cultivation method had a significant effect on the overall amount of acids, ketones, aldehydes and monoterpenes emitted by fruit, being the latter almost absent in organic fruit. IPM fruit had a generally higher VOCs emission and the volatilome was dominated by terpenes accounting for half of the total VOCs emission. Hexanal, 5-methylfurfural, nonanol, pentan-2-one, 2β-pinene, α-terpinene, β-myrcene, β-phellandrene, γ-terpinene, *o*-cymene, Z-3-heptene and E-3-heptene were not detected in organic fruits, whereas nonanal, 1-pentanol, acetoin, isoamyl acetate, β-caryophyllene, α-humulene and octanoic acid were missing in IPM grown berries. Although present in both treatments, limonene, β-ionone, 2-nonanone and octanal were more intensely emitted in IPM samples, whereas the emission of 1-octyl acetate, 2-heptanol and *trans*-geraniol was higher in organic fruits. PCA performed on the relative emission of single VOCs showed a clear separation of organic and IPM fruit volatilomes along components 1 and 2, explaining 85% and 9% of the total variance, respectively (Figure 6a). The four most important VOCs responsible for data clustering were α- and β-phellandrene, pentan-2-one and β-caryophyllene (Figure 6b).

**Figure 7:**
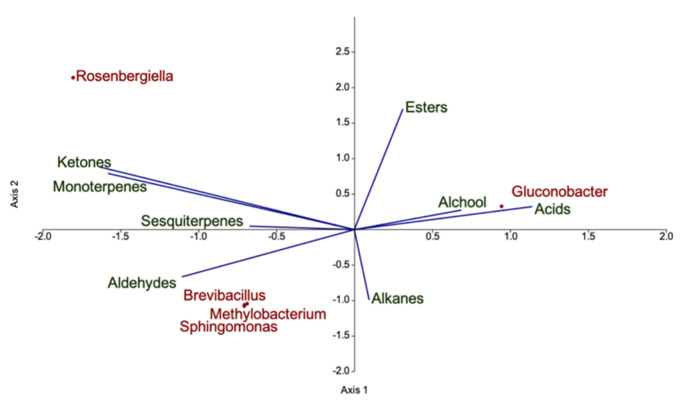
Canonical correlation analysis of bacterial genera discriminating organic from IMP grown fruit and volatile organic compounds classes. Bacterial genera are coloured in brown, volatile organic compounds classes in green.

Canonical correlation analysis was performed to investigate correlation between VOC classes and key bacterial genera (Figure 7). Bacterial genera abundance in organic and IPM fruits was averaged and a threshold of 3% was imposed to select key bacterial genera. *Gluconobacter* correlated with acids and alcohols, *Brevibacillus*, *Methylobacterium* and *Sphingomonas* genera were associated to aldehydes, whereas *Rosenbergiella* correlated with ketones and monoterpenes.

### 3.4. In Silico Fruit Volatilome Assembly

**Figure 8:**
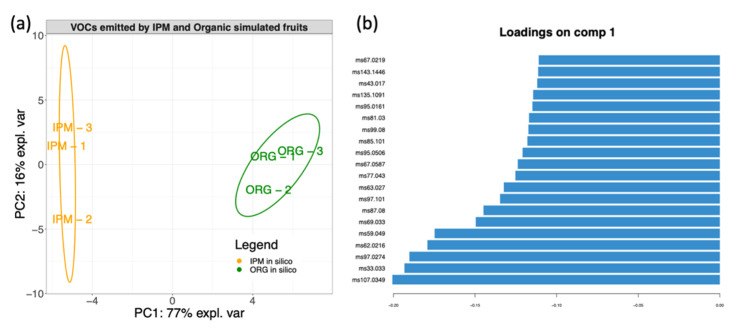
Characterization of volatile organic compound profiles, produced by assembling the emissions of single bacterial isolates from organic or IPM grown raspberries. Diagram shows (**a**) Principal Component (PC) 1 and 2 of principal component analysis (PCA) of the simulated fruits. Ellipses enclose confidence level = 95%. (**b**) loadings of the 20 *m*/*z* fragments showing highest correlation with PC1 in the PCA analysis.

To estimate the role of bacterial organic volatile compounds (bVOCs) on fruit volatilome, the emission of bVOCs by the bacterial species isolated from raspberry fruit was analysed by PTR-MS (Table S2). To mimic the fruit environment, bacterial isolates were grown on filter sterilized raspberry juice. The *m*/*z* emissions of each bacterial isolate, weighted by the relative abundance of its genus calculated from NGS data, were used to construct an in silico-assembled bacterial volatilome for organic and IPM grown fruit. PCA showed a clear discrimination of organic and IPM berries according to the volatilome of fruit associated bacterial community (Figure 8a). To identify the most relevant bVOCs among the 118 masses of the dataset, the loading values of Principal Component 1 (77% of explained variance) were extracted. The *m/z* fragments that showed the highest loading values were *m*/*z* 107.0349, *m*/*z* 33.033*, m*/*z* 97.0274, *m*/*z* 62.0216, *m*/*z* 59.049, *m*/*z* 69.033 and *m*/*z* 87.08. *m*/*z* 33.033, *m*/*z* 69.033, *m*/*z* 97.024 and *m*/*z* 107.0349 were putatively identified as methanol, furan, furfural and benzaldehyde, respectively, whereas *m*/*z* 59.049 and *m*/*z* 87.08 may attributed to linear aldehydes and ketones (C3 and C5, respectively) (Figure 8b).

## 4. Discussion

### 4.1. Diversity of Fruit-Associated Microbiomes

Several studies demonstrated the influence of management practices on soil microbial composition and functions [52]. In recent years, the microbial communities in the phyllosphere have gained increasing attention [53]. Nonetheless, only a few studies have focused on the microbial communities hosted by fruits grown with different cultivation methods, mainly with the purpose of assessing risks to human health [29] or managing post-harvest crop storage [54].

In this work, NGS analysis highlighted that IPM raspberry fruits were characterized by a higher bacterial biodiversity in comparison to organically grown ones, with *Brevibacillus* (28% relative OTU abundance), *Methylobacterium* (12%), *Rosenbergiella* (18%) and *Sphingomonas* (5%) being the most abundant genera. *Rosenbergiella* and *Sphingomonas* spp. were almost absent in organic fruits. *Rosenbergiella* belongs to the family Enterobacteriaceae and has been detected in several plant species [55]. *Sphingomonas* has been found in natural environments, such as soil and plants, and has been proven to possess multifaceted positive functions [56].

The bacterial community of organic berries was dominated by the genus *Gluconobacter* (73%), followed by *Brevibacillus* (14%) and *Methylobacterium* (4%) (Figure 1). *Gluconobacter* was only found on organic fruits. This genus includes acetic acid bacteria known to live on a wide variety of fruits, and encompasses saprophytic, symbiotic, and pathogenic species [57]. In particular, some *Gluconobacter* strains are known to cause postharvest fruit losses, by causing rotting and browning [58].

Farming practices such as fertilizer or pesticide application shape microbiome compositions by modifying nutrient availability and ecological niches [59]. Although different research works suggest that organic cultivation promotes microbial biodiversity in the soil and on fruit surface [29,60,61], our results indicate that, in raspberry, IPM cultivation promoted bacterial biodiversity on fruit. It can be hypothesized that in crop systems with low inputs and disturbances, such as that of organic farming, microbial communities are dominated by few, highly adapted bacterial species able to outcompete potential generalist colonizers, thus becoming predominant. Indeed, the natural habitats of *Gluconobacter* spp., the prevalent bacterial genera on organic fruits, are flowers and fruits [62].

On the other hand, chemical pesticide application affects microorganisms in complex communities, opening ecological niches otherwise occupied by competing microorganisms [63], with microbial community dynamics depending on the chemical nature, rates and frequency of treatments.

### 4.2. Quality Parameters of Organic and IPM Raspberry Fruits

Several studies report the influence of the cultivation method on the quality of fruits and berries [64,65,66]. As raspberry is a highly perishable product, the identification of the optimal maturity stage is crucial for fruit quality at consumption. Visual assessment of fruit colour or ease of fruit detachment from the receptacle are the parameters mostly used in raspberry industry to determine harvesting time. In this work, organic and IPM fruits were harvested at the same levels of detachment force, but they differed for several qualitative parameters (Figure 3). IPM fruits were characterized by higher weight and titratable acidity, whereas organic ones contained more anthocyanidins. During the raspberry maturation process, acidity shows a steady increase in the early stages, followed by a substantial decrease during ripening. Anthocyanidins content and fruit weight increase during maturation. Finally, variations in soluble solid content are negligible at the maturation stages close to harvest (i.e., physiological maturity) [4]. Therefore, the differences observed in the quality parameters may be related to a more advanced ripening of organic berries than IPM ones, in spite of similar detachment forces.

Exogenous IAA application on berries has been observed to stimulate ethylene emission, thus promoting fruit ripening [67]. IAA-producing bacteria, such as *P. agglomerans* and *T. terrea*, could be isolated only from organic fruit (Table 2). In this view, the difference in maturation could be related to the bacteria residing on the fruit, or on the fruitlet during development. An increase in ethylene production and respiration rate has been detected from the white fruit stage until full maturity [67,68] and induction of anthocyanin formation was promoted after ethylene application [69]. We suggest that the promotion of maturation by IAA producing bacteria exceeded the negative effect of auxin on anthocyanin content. In fact, IAA has been reported to have a negative correlation with anthocyanin contents indicating that anthocyanin accumulation starts when IAA content decreases [70]. Moreover, in raspberry, exogenous application of a high concentration of IAA (100 μM) at fruit with equal ripening stage was found to depress anthocyanins accumulation [71]. However, previous studies highlighted that organic cultivation promote anthocyanin content in berries [64,66,72,73]. Thus, other factors may have contributed to the increase of anthocyanin. It is generally assumed that organic plants contain more secondary metabolites, as a form of protection against the stress factors remediable in IPM crops by cultural inputs, i.e., fertilizers and pesticides [74]. In horticultural plants, an increase of total nitrogen generally depresses anthocyanin accumulation [75,76]. However, in strawberry cell culture, the percentage of NH_4_^+^ in the total nitrogen concentration directly correlated with changes in cell density and anthocyanin content [77]. Similarly, in purple basil, NH_4_^+^ availability positively correlated with the contents of anthocyanins [78]. All the bacterial species uniquely isolated in organic fruit can produce NH_4_^+^. Thus, the higher availability of this cation in fruits might have increased anthocyanin in organically produced berries.

Fruit acidity is an important component of organoleptic quality [79] and in berries it is influenced by several environmental and plant factors (i.e., plant age and genotype, temperature, harvest season and orchard location) [21]. In previous studies on raspberry ‘Tulameen’ and ‘Kweli’, higher titratable acidity values were found in organic raspberries versus IPM grown ones [73]. Contrastingly, our results highlighted lower acidity values in organic fruits. Several studies suggest a role of bacteria on fruit acidity. *Bacillus* sp. inoculated on flowers and leaves of sour cherry affected sugar content and titratable acidity of fruits [80]. Similarly, application of *Pseudomonas fluorescens* on strawberry plantlets altered fruit acidity [81]. In our work, canonical correspondence analysis reveals possible interaction between titratable acidity of fruits and *Methylobacterium* and *Sphingomonas* genera (Figure 4), more abundant in IPM fruits. *Methylobacterium* spp. is mainly found on the phyllosphere where it is able to use methanol as the sole carbon source, whereas several *Sphingomonas* spp. strains have recently gained attention for their ability to produce gibberellins [56,82,83], together with ACC deaminase-mediated reduction of ethylene in the host [84]. The particular combination of hormones may delay late ripening processes, such as degradation of organic acids. Additionally, the direct contribution of *Methylobacterium* or *Rosenbergiella* spp. [85,86] to fruit organic acid content cannot be ruled out.

For raspberries, the increase of fruit weight is an important factor to reduce production costs, especially labour [87]. Although plant nutrition is one of the main factors directly influencing berry weight [21], application of plant growth promoting bacteria on fruits also contributed to the increase of berry weight and size [81]. In our study, fruit weight was significantly higher in IPM fruits and correlated to *Brevibacillus* and *Rosenbergiella* abundance (Figure 4). *Brevibacillus* spp. has been recorded in very diverse environmental habitats and several strains successfully promoted plant growth [88]. For instance, application of *Brevibacillus* spp. on eggplant and pepper led to an increased number of fruits per plant as well as fruit size, weight, and yield [89]. *Rosenbergiella* is a novel genus initially identified in floral nectar of almond [90], but it has been found in the flowers of several plant species [86], pollen and insects associated with the flower [91]. This bacterium has been isolated also from fruit pericarp [92], suggesting that its population on the flower could be transmitted also on fruit during development and possibly to seeds [93]. In seeds, *Rosenbergiella* seems to promote germination by the production of IAA [94]. Although the *Rosenbergiella* spp. strain, isolated in this work from fully ripe fruit, did not show IAA production, this genus is known to include IAA-releasing species which may promote plant and fruit growth [95]. IAA has also an important role in fruit development which was demonstrated by the overexpression of the auxin synthesis-related gene (DefH9-iaaM) in transgenic plants, that showed increased fruit size and number [96]. Altogether, the observed differences in fruit quality, size and yield may originate indirectly as a result of bacterial influence on plant growth and fitness at different phenological stages or on different organs.

### 4.3. Volatilome and Bacterial Contribution to Fruit Aroma

Raspberry aroma is a key component for consumers’ perception of sensory quality [5]. Monoterpenes are the largest class of compounds, among which terpinen-4-ol, geraniol, linalool, limonene, nerol, *p*-cymene, terpinolene, α- and β-phellandrene, γ-terpinene and α- and β-pinene are the most frequently reported [5]. However, only few of the several VOCs identified in the literature are recognized as important descriptors of raspberry aroma, namely 4-(4-hydroxyphenyl)butan-2-one (also known as raspberry ketone), which contributes to the pure raspberry aroma; α-ionone and β-ionone (whose odour is described as violet-like), which are responsible for the overall fruit aroma [97]. In this work, raspberry ketone could not be detected because its linear retention index (LRI) lies outside the detection limit of this work.

Interestingly, IPM fruits were richer in β-ionone and 5-methylfurfural. The first compound is characterized by sweet and fruity odour, with seedy nuances, whereas the second one typically has almond and cherry notes. These VOCs possibly conferred more intense floral and fruity notes to the aroma of IPM fruits, which were also characterized by the presence of hexanal. The presence of this compound, a green-odour leafy compound, conferring grassy notes to fruit aroma, corroborates the hypothesis that IPM strategy delayed ripening of berries. A higher content of 2-heptanol and *trans*-geraniol in organic raspberries was observed. Interestingly, both compounds are known for being emitted by flowers of many species and to be effective insect repellents [98,99], thus suggesting that they have been produced as a defence-related response in organic fruit where pest attacks are less controlled. During post-harvest handling, raspberry fruits are highly susceptible, to fungal diseases, in particular grey mould caused by *B. cinerea* [100]. Remarkably, in this work IPM fruits were lacking β-caryophyllene, which is one of the volatile organic compounds proven to lower the susceptibility of fruits to *B. cinerea* [100]. Identification of VOCs present in raspberry fruits have been recently extensively reviewed [5]. In addition to VOCs previously described, we observed the presence of tetradecanal, 3-heptene isomers and 1-octyl acetate. The latter was found to be emitted in a significant higher amount in basil plants treated with NaCl [101]. In our work, this compound was significantly more abundant in organic raspberries which might suggest that these plants underwent and seek to react to a more stressful condition with respect to IPM ones. Overall, these results might be explained by the lack of use of pesticides and fertilizers in the organic management. Indeed, these conditions might stimulate plant self-protection mechanism and promote the synthesis of secondary metabolites in plants and fruits [102].

Bacteria produce VOCs that act as direct plant-protectants against pathogens, induce plant defences or promote plant growth and nutrition [103]. Here, we observe a difference in the microbiome and volatilome of raspberry fruits originating from different cultivation methods. Although demonstrating the bacterial origin of a particular volatile compound requires dedicated research, it is interesting to note that emission of acids by raspberry fruits is highly correlated to the presence of *Gluconobacter* bacteria (Figure 7). In our work, among the volatile compounds belonging to the acids, part of the acetic acid emitted by fruits might be of bacterial origin. Indeed, *Gluconobacter* spp. is known to colonize sugar-rich niches (such as fruit skin) and to efficiently convert sugars to acetic acid [104]. *Rosenbergiella* is a distinctive and abundant colonizer of IPM grown raspberries, here correlated with ketones and monoterpenes emissions. The draft genome of *R. nectarea* suggests that this species may affect fruit quality by releasing terpenoids and by degrading pectins [86], thus confirming a possible role in volatile emission diversity. *Methylobacterium*, *Brevibacillus* and *Sphingomonas* are more abundant in IPM grown fruits and positively correlate with aldehydes emission. Among these bacterial genera, *Methylobacterium* (in particular *M. extorquens*) has been thoroughly studied for its ability to enhance strawberry flavour [105,106]. In fact, *M. extorquens* possesses alcohol dehydrogenase enzymes capable of oxidizing a wide range of alcohols to aldehydes and ketones.

Volatiles produced by bacterial isolates on raspberry juice were used for the assembly of in silico fruit volatilome of organic and IPM fruits. Although authors are aware of the limitations imposed by such an artificial system, a clear separation among VOC typical of microbiomes of differently grown berries was observed (Figure 8). Fragments of *m*/*z* 59.049, 87.08, 97.0274 and 107.0349 were identified as belonging to the aldehyde or ketone class, and were typical of the IPM in silico volatilome. Interestingly, this result is in accordance with volatile emissions of the real fruits and, together with correlations between microbiome and VOC emissions, pave the way for further studies about the role of bacteria in shaping raspberry aroma.

## 5. Conclusions

This work provides evidence of the effects of organic and IPM cultivation methods on quality and aroma of raspberry fruits, and relates them with the cultivation-mediated changes in fruit-associated bacterial microbiome, supporting the hypothesis of a contribution of the fruit-associated microflora on raspberry quality traits. Organic farming practices were associated to lower bacterial biodiversity of fruit epiphytic microbiome, and resulted in smaller fruits with higher anthocyanin content and lower titratable acidity with respect to IPM fruits. Additionally, IPM fruits were characterized by a higher emission of sweet and fruity odours. Although the manipulation of fruit microbiome composition by cultivation methods requires further research, a higher microbial diversity plausibly determines a more complex, and overall more pleasant fruit aroma, and its enhancement may positively impact on raspberry quality. The involvement of bacteria in fruit development and quality was corroborated by known hormonal and metabolic activities of the microbial species isolated or recognized in the genera identified by NGS analysis. Some bacterial genera, including *Gluconobacter*, *Sphingomonas*, *Rosenbergiella*, *Brevibacillus* and *Methylobacterium* are suggested to contribute to fruit quality, and to enable the control of certain aspects of raspberry.

## Figures and Tables

**Table 1:** Bacterial genera and species isolated from raspberry fruits ‘Enrosadira’ cultivated with organic or IPM practices.

Fruit Type	Bacterial Isolate
*Both IPM and Organic*	*Bacillus subtilis,* *Burkholderia* spp., *Cellulomonas* spp., *Enterobacter asburiae,* *Enterobacter* spp.—strain 1, *Erwinia aphidicola,* *Erwinia rhapontici,* *Erwinia toletana,* *Lactobacillus plantarum,* *Methylobacterium extorquens,* *Ochrobactrum intermedium,* *Ochrobactrum pseudogrignonense,* *Paenibacillus alvei,* *Paenibacillus macerans,* *Paenibacillus polymixa,* *Pantoea agglomerans*.—strain 1, *Pantoea ananatis,* *Pantoea rwadensis,* *Pseudomonas fluorescens*—strain 1, *Pseudomonas fluorescens*—strain 2*,* *Pseudomonas stutzeri,* *Sphingomonas camponoticapitis,* *Sphingomonas paucimobilis,*
*IPM*	*Rosenbergiella* spp. *Enterobacter* spp—strain 2 and 3
*Organic*	*Pantoea agglomerans—*strain 2 *Tatumella punctata* *Tatumella terrea* *Cronobacter* spp. *Klebsiella oxytoca*

**Table 2:** Functional traits of bacteria strains isolated uniquely in organic or IPM berries.

Cultivation Method	Bacterium	IAA Production	Acetoin Production	NH_4_^+^ Production	Siderphores Production	ACC Deaminase Activity
IPM	*Rosenbergiella* spp.	-	-	-	-	-
	*Enterobacter* strain 2	-	+	-	+	-
	*Enterobacter* strain 3	-	-	+	+	-
ORG	*Pantoea agglomerans* strain 2	++	-	+	-	-
	*Cronobacter* spp.	-	-	+	-	-
	*Klebsiella oxytoca*	-	-	+	+	-
	*Tatumella punctata*	-	-	+	-	-
	*Tatumella terrea*	++	-	+	-	-

Qualitative rating of compound production/activity: − = not detected, + = detectable, ++ = high.

**Table 3:** Volatile organic compounds emitted by raspberry fruit ‘Enrosadira’ cultivated with organic (Org) or integrated (IPM) practices. Data are reported as mean ± SE. Values labelled with different letters indicates significant differences between the cultivation methods according to Student’s *t*-test at *p*-value < 0.05. nd = not detected.

Class	Compound	RetentionTime (min)	Linear Retention Index	Mean of Volatile Compounds Concentration (1-Octanol Equivalents)	
				Org	IPM
Aldehydes	(E)-2-Hexenal	11.89	1192	0.25 ± 0.25 ^a^	6.82 ± 2.35 ^a^
	Hexanal	5.34	1067	nd	5.65 ± 0.75
	Octanal	15.95	1274	0.47 ± 0.07 ^b^	0.76 ± 0.11 ^a^
	Tetradecanal	37.71	1942	0.63 ± 0.14 ^a^	0.44 ± 0.22 ^a^
	5-Methylfurfural	27.51	1578	nd	0.81 ± 0.03
	Nonanal	20.46	1376	0.45 ± 0.03	nd
Alchools	6-Methyl-1-heptanol	18.11	1319	0.06 ± 0.03 ^a^	0.03 ± 0.03 ^a^
	1-Pentanol	14.27	1241	0.14 ± 0.07	nd
	2-Nonanol	29.29	1508	0.10 ± 0.01 ^a^	0.05 ± 0.05 ^a^
	2-Heptanol	17.82	1312	0.84 ± 0.14 ^a^	0.43 ± 0.08 ^b^
	β -Linalool	26.13	1535	0.55 ± 0.14 ^a^	0.05 ± 0.05 ^a^
	Isogeraniol	40.33	2039	0.21 ± 0.11 ^a^	0.33 ± 0.24 ^a^
	Nonanol	29.56	1645	nd	0.18 ± 0.09
	Trans-geraniol	34.71	1827	0.99 ± 0.08 ^a^	0.67 ± 0.09 ^b^
Ketones	Acetoin	15.23	1259	0.31 ± 0.11	nd
	2-Heptanone	9.99	1136	0.60 ± 0.20 ^a^	0.45 ± 0.16 ^a^
	2-Nonanone	20.27	1372	0.38 ± 0.10 ^b^	1.08 ± 0.27 ^a^
	2-Undecanone	27.58	1581	0.18 ± 0.04 ^b^	1.15 ± 0.24 ^a^
	Pentan-2-one	3.91	1012	nd	7.35 ± 2.35
Esters	1-Octyl acetate	23.71	1463	3.05 ± 0.19 ^a^	0.75 ± 0.15 ^b^
	Ethyl hexanoate	13.47	1225	0.71 ± 0.08 ^a^	2.26 ± 1.13 ^a^
	Isoamyl Acetate	6.97	1130	0.84 ± 0.24	nd
	Methyl Salicylate	32.75	1756	0.66 ± 0.33 ^a^	1.02 ± 0.31 ^a^
Sesquiterpenes	β-Caryophyllene	27.21	1569	5.48 ± 1.38	nd
	α-Humulene	29.39	1640	1.34 ± 0.43	nd
	α-Ionone	34.5	1819	13.99 ± 1.16 ^a^	13.35 ± 0.80 ^a^
	β-Ionone	36.79	1906	19.86 ± 1.97 ^b^	35.00 ± 2.49 ^a^
	Dihydro α-ionone	33.59	1786	1.57 ± 0.09 ^a^	0.96 ± 0.24 ^b^
	Dihydro β-ionone	34.05	1802	5.63 ± 0.86 ^a^	6.53 ± 1.33 ^a^
Monoterpenes	2-Carene	15.47	1264	0.07 ± 0.03 ^a^	0.38 ± 0.24 ^a^
	2-β-Pinene	6.19	1092	nd	0.54 ± 0.16
	α-Phellanderene	8.79	1124	0.01 ± 0.01 ^a^	15.33 ± 4.72 ^a^
	α-Terpinene	9.65	1133	nd	0.86 ± 0.43
	β-Myrcene	9.18	1128	nd	4.91 ± 1.90
	β-Phellandrene	11.32	1177	nd	9.65 ± 2.96
	γ-Terpinene	13.65	1229	nd	0.67 ± 0.37
	Limonene	10.85	1164	0.08 ± 0.01 ^b^	2.54 ± 0.79 ^a^
	o-Cymene	14.81	1251	nd	2.69 ± 0.90
Acids	Acetic acid	22.28	1424	2.38 ± 0.62 ^a^	1.02 ± 0.22 ^a^
	Nonanoic acid	42.45	2119	0.41 ± 0.18 ^a^	0.08 ± 0.08 ^a^
	Octanoic acid	39.94	2025	0.78 ± 0.15	nd
Alkanes	Tridecane	17.38	1302	7.02 ± 3.55 ^a^	0.02 ± 0.02 ^a^
	Z-3-Heptene	7.55	1131	nd	2.34 ± 0.58
	E-3-Heptene	7.77	1113	nd	1.89 ± 0.56

## Data Availability

Data is contained within the article or Supplementary Material.

## Supplementary Materials

The following are available online at https://www.mdpi.com/article/10.3390/microorganisms9081617/s1, Figure S1: Rarefaction curves of community richness estimates of IPM (Conv) and (Organic) raspberry samples. Table S1: Diversity indexes for IPM and organic raspberry samples. Table S2: Emission (mean ± SE) and tentative identification of *m*/*z* fragments detected by proton-transfer reaction−mass spectrometry (PTR-MS) analysis of raspberry juice cultures of bacterial isolates, and loadings of each *m*/*z* fragment on principal components 1, 2 and 3 of the principal component analysis (PCA) built on in silico fruit emissions. Nd = not detected.
