# Supplementary material for: Does Organic Farming Increase Raspberry Quality, Aroma and Beneficial Bacterial Biodiversity?
Source: Microorganisms. 2021 Jul 29;9(8):1617. doi: 10.3390/microorganisms9081617 (PMC8400319; doi:10.3390/microorganisms9081617)
Supplement: Supplementary file 1 [file microorganisms-09-01617-s001.zip › Supplementary Material.pdf]

Supplementary material

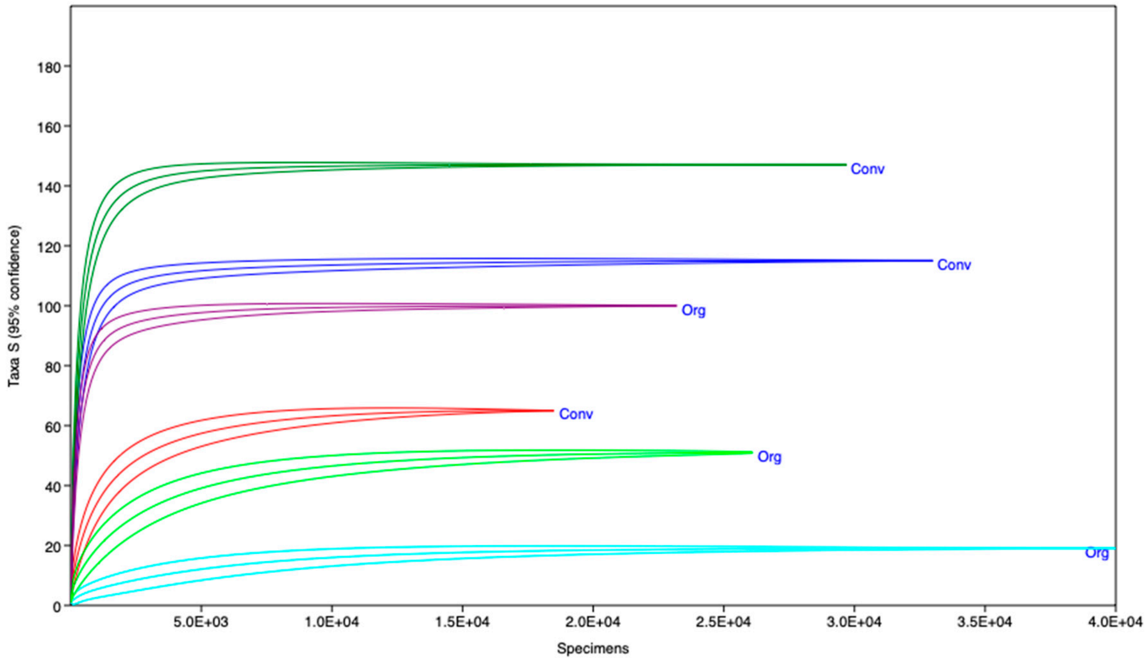

Figure S1. Rarefaction curves of community richness estimates of IPM (Conv) and (Organic) raspberry samples.

Table S1. Diversity indexes for IPM and organic raspberry samples.

	Organic	*IPM*
Taxa (S)	147	269
Individuals (n)	29955	27216
Simpson (1-D)	0.7272	0.9146
Shannon (H)	2.008	3.626
Equitability (J)	0.4024	0.6481
Chao-1	154.5	282

Table S2. Table S2: Emission (mean ± SE) and tentative identification of *m*/*z* fragments detected by PTR-MS analysis of raspberry juice cultures of bacterial isolates, and loadings of each *m*/*z* fragment on principal components 1, 2 and 3 of the PCA built on in silico fruit emissions. Nd = not detected. See attached excel file.
